# Effectiveness of base‐of‐skull immobilization system in a compact proton therapy setting

**DOI:** 10.1002/acm2.12323

**Published:** 2018-04-06

**Authors:** Ghazal Shafai‐Erfani, Twyla Willoughby, Naren Ramakrishna, Sanford Meeks, Patrick Kelly, Omar Zeidan

**Affiliations:** ^1^ Department of Radiation Oncology Orlando Health – UF Health Cancer Center Orlando Orlando FL USA

**Keywords:** base of skull, immobilization, intracranial tumors, localization accuracy, proton therapy

## Abstract

**Purpose:**

The purpose of this study was to investigate daily repositioning accuracy by analyzing inter‐ and intra‐fractional uncertainties associated with patients treated for intracranial or base of skull tumors in a compact proton therapy system with 6 degrees of freedom (DOF) robotic couch and a thermoplastic head mask indexed to a base of skull (BoS) frame.

**Materials and methods:**

Daily orthogonal kV alignment images at setup position before and after daily treatments were analyzed for 33 patients. The system was composed of a new type of thermoplastic mask, a bite block, and carbon‐fiber BoS couch‐top insert specifically designed for proton therapy treatments. The correctional shifts in robotic treatment table with 6 DOF were evaluated and recorded based on over 1500 planar kV image pairs. Correctional shifts for patients with and without bite blocks were compared.

**Results:**

Systematic and random errors were evaluated for all 6 DOF coordinates available for daily vector corrections. Uncertainties associated with geometrical errors and their sources, in addition to robustness analysis of various combinations of immobilization components were presented.

**Conclusions:**

Analysis of 644 fractions including patients with and without a bite block shows that the BoS immobilization system is capable of maintaining intra‐fraction localization with submillimeter accuracy (in nearly 83%, 86%, 95% of cases along SI, LAT, and PA, respectively) in translational coordinates and subdegree precision (in 98.85%, 98.85%, and 96.4% of cases for roll, pitch, and yaw respectively) in rotational coordinates. The system overall fares better in intra‐fraction localization precision compared to previously reported particle therapy immobilization systems. The use of a mask‐attached type bite block has marginal impact on inter‐ or intra‐fraction uncertainties compared to no bite block.

## 1 INTRODUCTION

Patient immobilization is critical to the safe and accurate delivery of radiation therapy. This is especially critical in particle therapy because of strong dependence of beam range on even the smallest variation in patient position with respect to the reference conditions.^1^, ^2^ Thus, it is important to minimize uncertainties associated with patient motion by understanding limitations of in‐room immobilization systems and their performance throughout the course of treatment. Accurate characterization of immobilization system performance can provide useful data for physicists and physicians for determining planning margins, which have significant impact on healthy tissues and organs at risk.

Most commercially available head immobilization devices for proton therapy use a relatively thin layer of carbon fiber composites compared to more rigid construction of their x ray therapy counterparts.^1^, ^2^ Moreover, to maintain sharp lateral penumbra, air gap between the aperture and patient needs to be minimal. Therefore, the use of bulky immobilization devices is not desired because they potentially increase likelihood of collision scenarios. It is typically challenging to satisfy two conflicting goals of maintaining rigidity and minimizing material in the beam path. A careful balance of these requirements is attained in the design of immobilizing frames such as the widely used base‐of‐skull (BoS) carbon‐fiber frame (kVue^TM^ BoS insert) by Qfix (Avondale, PA, USA) which is evaluated in this study. Despite the widespread use of BoS frame in proton centers, there are few reports evaluating its effectiveness for immobilization with 6 DOF robotic couches.^3^

The purpose of this study is to analyze daily repositioning accuracy of patients treated for intracranial or base of skull tumors in a compact proton therapy system. For this reason, we evaluate inter‐ and intra‐fractional uncertainties associated with patient movement throughout the treatment. Furthermore, we investigate the effect of utilizing a bite block on inter‐ and intra‐fractional uncertainties. To our knowledge, this is the first study of this kind on a compact proton system setting such as the Mevion S250 system.

## 2 MATERIALS AND METHODS

Our clinic is equipped with a Mevion S250 compact proton therapy system (Mevion Medical Systems, Littleton, MA, USA) accelerator with 190° rotating gantry and a 270‐degree robotic couch (KUKA Roboter, GmbH, Augsburg, Germany) with 6 DOF capable of executing submillimeter translational motions and rotational motions. Robotic couches with 6 DOF are known for their motion accuracy and precision^4^, ^5^ which is why they are primarily used for proton therapy. Hsi et al.^6^ reported residual target displacement for KUKA robotic couch rotations with respect to isocenter to be within 0.5 mm and nearly 85% of all couch movements were within 0.5 mm in the horizontal plane and within 0.7 mm vector distance from required displacements. The robotic couch in this study is capable of maintaining the position of the isocenter within submillimeter accuracy throughout the treatment and this accuracy is routinely verified on a monthly basis during Monthly QA.

**Figure 1:**
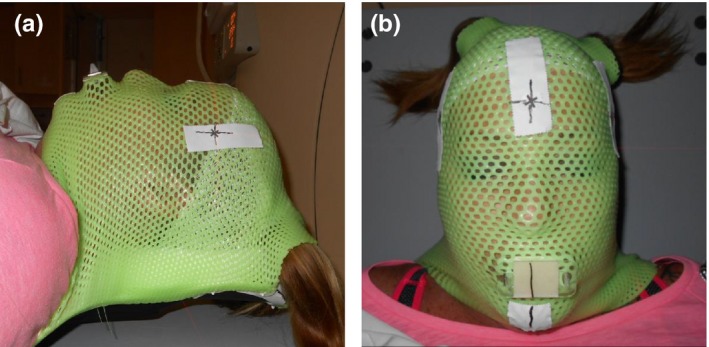
Lateral (a) and anterior (b) views of a patient in a Klarity mask with a bite block. Patient's head is rested on a Klarity cushion, and the mask is attached to a BoS frame.

Patient immobilization was achieved using a BoS frame insert with a new type of aquaplast mask and a cushion by Klarity (Newark, OH, USA) as shown in Fig. 1. This new mask is known for its nonstick properties and relatively slow hardening process, which make it easier to prepare and conform to patient's anatomy. The cushion was used to support posterior skull in a comfortable and reproducible position. For certain patients, and per physician directive, a mask combined with a Klarity bite block with malleable thermoplastic material was used with goal of decreasing patient motion within the mask. Subsequently, the bite block was attached to the mask and the mask was indexed to the BoS frame.

**Figure 2:**
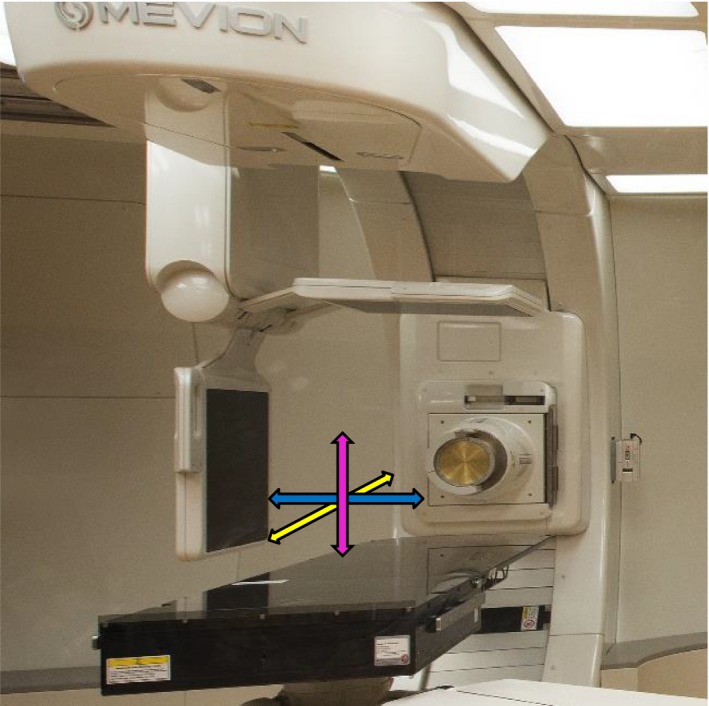
The Mevion S250 Double Scattering system illustrating compact gantry design with small applicator and the robotic couch in setup position. The snout is shown at 90° position. x, y, and z axes are shown in yellow, blue, and magenta, respectively. Orthogonal kV imagers are in imaging position. Translational corrections along x, y, and z are described as SI, LAT, and PA, respectively. Rotational corrections around x, y, and z axes are referred to as roll, pitch, and yaw, respectively.

Once the patient was simulated, treatment was planned in Pinnacle^3^ (Phillips Radiation Oncology Systems, Fitchburg, WI, USA) treatment planning system (TPS). Setup couch position was calculated based on the position of the fiducial markers embedded in the CT couch‐top, which is identical to the treatment couch‐top. For first treatment fraction, the robotic couch brought the patient to this pre‐calculated position. The Radiation Therapy Technologists (RTTs) verified that the lasers coincide with external markers before they move the patient to the isocenter location based on shifts from the treatment plan. Final localization was achieved through acquisition of orthogonal X‐ray pairs (PA and LAT) at setup position (couch position 270° as shown in Fig. 2). Couch coordinates at the final position were saved and used thereafter as the starting point for daily treatments. The RTTs compare daily‐acquired images at setup position against digitally reconstructed radiographs (DRRs) from reference CT and manually align them based on bony‐anatomy within the Verity^TM^ software (Mevion Medical Systems). Alignments were performed to patient‐specific anatomical structures based on physician instructions. For every fraction of the treatment, the corresponding correctional shifts from the nominal position were sent to the robotic couch. Once applied, the final images and shifts were recorded in the MOSAIQ record and verification system (Elekta Inc, Atlanta, GA, USA). The robotic couch is then rotated by 90° from setup position to bring the patient to either the left or right lateral treatment position. To monitor immobilization robustness, post‐treatment imaging was performed after delivery of all treatment beams at the end of 84 fractions for 14 patients. Correctional shifts data were retrospectively extracted from MOSAIQ and analyzed in order to evaluate inter‐ and intra‐fractional motions. The latter provides valuable information on efficacy of performance of the immobilization technique. It is worth mentioning that motion accuracy for our robotic couch is consistent as long as isocenter is located inside the so‐called “Couch Treatable Volume”, which is a space that includes the treatment end of couch‐top and enough space above it in which a patient isocenter can be located. The treatable volume covers a rectangular solid volume which moves with couch‐top and has dimensions of 50 cm wide, 39 cm tall, and 95 cm long. Couch corrections that will put isocenter outside the Treatable Volume will not be executed in Verity^TM^ software.

In order to assess various components of uncertainties in the immobilization and localization system, we employ the approach adopted from van Herk's population based statistics.^7^ Below, the definition of each error category is presented:

- *M*_*g*_ *or Mean group error*:
  $$M_{g}=\sum_{i=1}^{N}d_{i}/N$$
  where $d_{i}$ is the average inter‐fractional displacement for a single patient *i* throughout the course of treatment, and N denotes the number of patients in the group under study.
- $\sigma_{g}$ *or systematic error*:
  $$\sigma_{g}=SD\left(d_{i}\right)$$
  where $SD(d_{i})$ is the standard deviation of the average inter‐fractional displacement measurements for each patient.
- $\sigma_{p}$ *or inter‐fraction random error*:
  $$\sigma_{p}=\sqrt{\sum_{i=1}^{N}SD_{\left(d_{i}\right)}^{2}/N}$$
  where $SD(d_{i})$ is the standard deviation of the average inter‐fractional displacement measurements for each patients.
- $\sigma_{f}$ or i*ntra‐fraction uncertainty*_:_ $\sigma_{f}$ is calculated using the same equation as $\sigma_{p}$, utilizing post‐treatment images.

Once the above uncertainties are assessed, appropriate margins are applied to ensure robust delivery of prescribed dose to target volume.

In this study, we analyze inter‐fractional translational displacements in addition to rotational alignments on 33 patients including five cases of meningioma, 12 cases of glioma, and seven cases of base of skull tumors among others. The inter‐ and intra‐fractional data were obtained for 644 and 84 fractions, respectively. Patients were further divided into two subgroups based on application of a bite block or lack thereof, and were analyzed to assess the efficiency of the bite block in immobilization of the patient.

**Figure 3:**
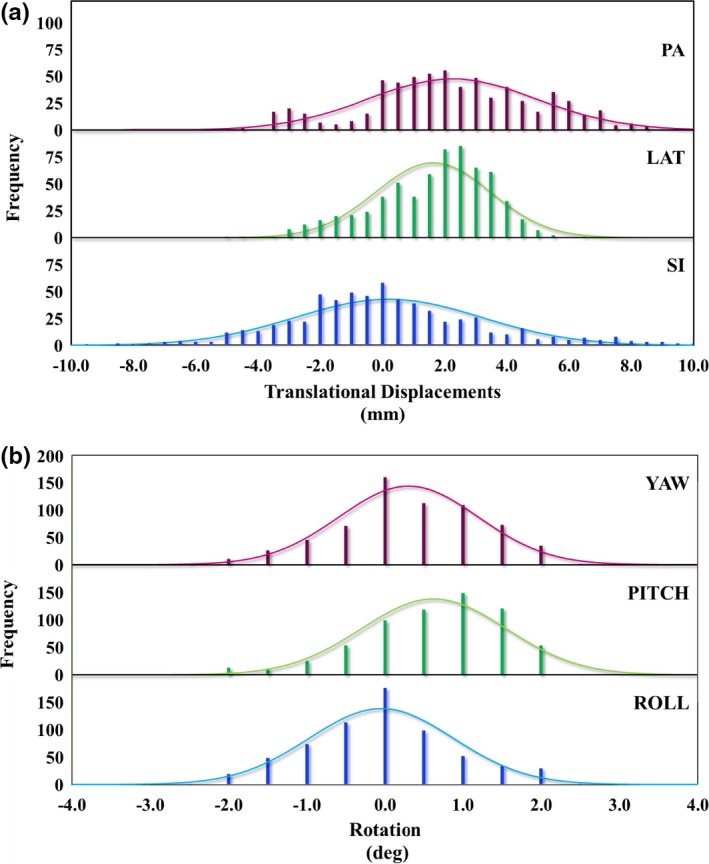
Histograms of inter‐fractional (a) translational displacements along three ordinal axes and (b) rotational displacements around three ordinal axes. Solid lines represent corresponding Gaussian distributions.

In order to further investigate the source of asymmetries shown by the Gaussian distributions in Fig. 3, we carried out phantom‐based image alignment measurements under different weight conditions. A Klarity mask was used to conform to the head and neck section of an anthropomorphic RANDO phantom (The Phantom Laboratory, Greenwich, NY, USA) on a BoS frame with the exact procedure that is used on a real patient. Several Bumper weight plates were placed along the treatment table to simulate the weight and overall weight distribution of patients. The mask was marked and aligned with lasers similar to a real patient. Two CT scans of the exact setup were performed, once for a total weight of 100 lbs (phantom plus Bumper weights) and once for a total weight of 200 lbs. For each set of DICOM images, respective couch positions were calculated based on placement of the isocenter in the RANDO brain volume and locating the tip of the BoS couch‐top on each scan. Coordinates of the couch at setup position were then calculated and sent to the robotic couch via MOSAIQ. For each weight, the phantom was set up in the treatment room ten times with the BoS frame to simulate 10 independent patient treatments. A set of orthogonal kV images were acquired for each setup and compared against DRRs and required shifts and angular corrections were recorded.

## 3 RESULTS

### 3.A Inter‐fractional translational and rotational displacements

Inter‐fractional translational displacements are obtained along three ordinal directions, namely x (superior‐inferior, SI), y (left‐right (or lateral), LAT), and z (posterior‐anterior, PA). Rotations around the above axes construct roll, pitch, and yaw, respectively. Histograms of the above distributions and statistical values associated with them are presented in Fig. 3 and Table 1. Kolmogorov–Smirnov goodness‐of‐fit test reveals that the above distributions are of Gaussian nature. To illustrate this clearly, we overlay a Gaussian fit for every histogram.

**Table 1:** Statistical values associated with distribution of inter‐ and intra‐fractional translational and rotational shifts for all patients

	Inter‐fractional						Intra‐fractional					
	Translational (mm)			Rotational (deg)			Translational (mm)			Rotational (deg)		
	SI	LAT	PA	ROLL	PITCH	YAW	SI	LAT	PA	ROLL	PITCH	YAW
Mean	0.3	1.6	2.3	0.0	0.6	0.3	−0.4	−0.1	0.3	0.0	0.0	0.0
MAX	11.6	6.3	8.4	2.4	3.1	2.3	1.5	1.7	1.6	1.0	1.3	1.5
MIN	−9.4	−4.8	−4.6	−2.5	−2.0	−2.7	−2.0	−2.6	−0.5	−1.5	−1.0	−1.8

### 3.B Intra‐fractional translational and rotational displacements

**Figure 4:**
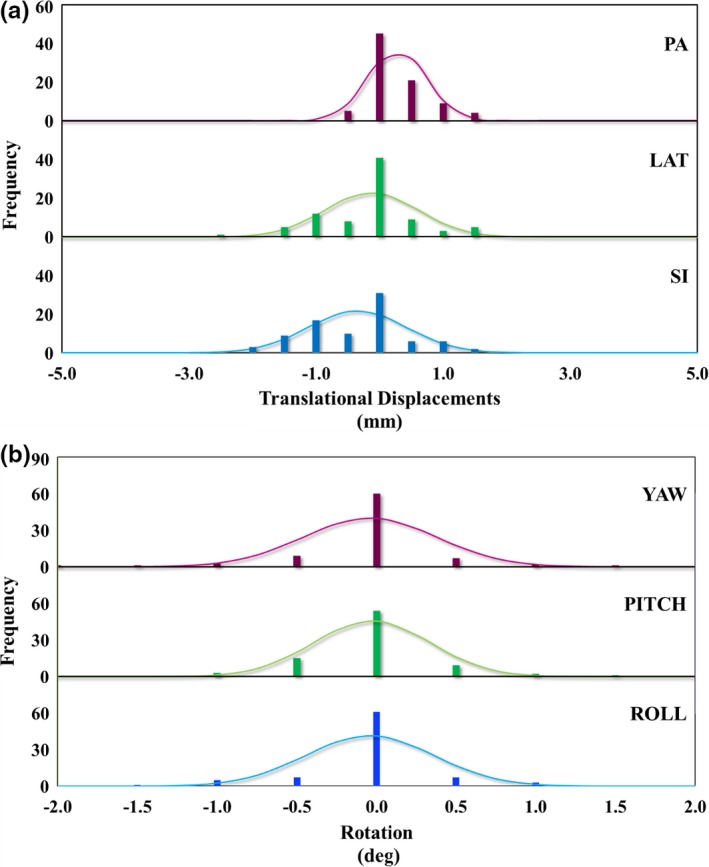
Histograms of intra‐fractional (a) translational displacements along three ordinal axes, and (b) rotational displacements around three ordinal axes. Solid lines represent corresponding Gaussian distributions.

Histograms of intra‐fractional translational and rotational displacements and statistical values associated with them are presented in Fig. 4 and Table 1, respectively. We find the magnitude of both translational and rotational displacements to be smaller and more symmetric for intra‐fractional motion compared to inter‐fractional displacements and all rotations show similar distribution profiles centered and symmetrical around zero.

### 3.C Evaluation of the inter‐ and intra‐fractional uncertainties

A summary of data analysis for all patients for inter‐ (n = 644) and intra‐ (n = 84) fractions is presented in Table 2. For all patients included in this study the highest value for M_g_ error was 2.3 mm along PA direction. M_g_ error for inter‐fractional motion along LAT direction was 1.6 mm. We found the systematic errors (*σ*_g_) along PA and SI direction to be higher (2.8 and 2.6 mm respectively) compared to 1.4 mm along LAT. Random errors (*σ*_p_) along these two axes follow the same pattern (1.0 and 0.4 mm, respectively). We found the intra‐fractional uncertainty along PA axis (0.4 mm) to be the highest compared to LAT and SI direction (both equal to 0.1 mm).

**Table 2:** Mean (M_g_), systematic (*σ*_g_), random (*σ*_p_) errors associated with translational and rotational inter‐fractional motion for all studied patients based on 644 fractions. Intra‐fraction uncertainty (*σ*_f_) is shown in the last row, and is based on 84 fractions

	Translational (mm)			Rotational(deg)		
	SI	LAT	PA	ROLL	PITCH	YAW
Mg	0.3	1.6	2.3	0.0	0.5	0.2
*σ*g	2.6	1.4	2.8	0.6	0.7	0.5
*σ*p	0.4	0.2	1.0	0.1	0.1	0.7
*σ*f	0.1	0.1	0.4	0.1	0.1	0.3

### 3.D Effectiveness of bite block in conjunction with Klarity mask

In this section, we divide the patients into two groups based on whether they had a bite block or not. Table 3 summarizes uncertainties for the corresponding groups. For the group for whom bite blocks were used, setup images from 328 fractions and post images from 50 fractions were analyzed. For the other group, setup images from 316 fractions and post images from 34 fractions were studied. Translational M_g_ along PA was 3.5 mm for the group with bite blocks and 0.9 mm for the group without one. *σ*_g_ is smaller along all directions for cases without bite blocks. The differences in rotational M_g_ and *σ*_g_ are negligible as seen in Table 3 indicating that the use of bite block does not improve overall immobilization. Translational and rotational *σ*_p_ and *σ*_f_ for every degree of freedom does not change for cases with a bite block compared to cases without one.

**Table 3:** Mean (M_g_), systematic (*σ*_g_), random (*σ*_p_) errors associated with translational and rotational inter‐fractional motion of two groups of patients treated with Klarity mask with or without a bite block. Analysis is based on inter‐fractional motion for 328 fractions (with bite block) and 316 fractions (without bite block), respectively. Intra‐fraction uncertainty (*σ*_f_) is shown in the last row, and is based on 50 fractions (with bite block) and 34 fractions (without bite block), respectively

	Klarity with bite block						Klarity without bite block					
	Translational (mm)			Rotational (deg)			Translational (mm)			Rotational (deg)		
	SI	LAT	PA	ROLL	PITCH	YAW	SI	LAT	PA	ROLL	PITCH	YAW
Mg	1.2	1.5	3.5	0.2	0.7	0.1	‐0.8	1.7	0.9	‐0.2	0.4	0.4
*σ*g	2.6	1.7	2.6	0.6	0.7	0.6	2.0	1.1	2.3	0.5	0.8	0.5
*σ*p	0.5	0.3	1.0	0.2	0.1	0.7	0.6	0.3	1.0	0.2	0.2	0.7
*σ*f	0.2	0.2	0.4	0.1	0.1	0.3	0.3	0.2	0.3	0.2	0.1	0.4

### 3.E Phantom measurements

Comparison of the 100 and 200 lbs scans revealed that the couch‐top in the simulation room sags due to increased relative weight on couch‐top. Once couch coordinates were calculated, the alignment images were analyzed. The mean magnitude of the couch shifts along PA direction was found to be 3.3 mm different between both weights with larger PA shift corresponding to the heavier weight. The mean of pitch was different for the two weights by 0.15°. It is worth noting that based on medical records, most of our patients weighed less than 150 lbs, which is in agreement with the direction of patient shifts that has been observed in this study as shown in Fig. 3. The observed displacement of the Gaussian distribution on lateral shifts seen in Fig. 3 is attributed to an inherent offset in lateral BoS frame position with respect to CT isocenter compared to treatment room isocenter. This systematic difference is due to hardware of the latching system of the base of the BoS frame with CT couch‐top and in‐room robotic couch base. The magnitude of this offset falls in the range of 1–2 mm and differs slightly between patients due to variables such as patient weight and the lateral force applied to the BoS frame during load and unloading of the patient. We verified this offset by recording pixel coordinates of the implanted BBs in the BoS frame based on CT images and in‐room images for several patients. Therefore, upon image‐based alignment of the patient in treatment room, a systematic lateral shift in the positive direction is needed to compensate for the aforementioned discrepancy. This positive shift is responsible for the lateral distribution to be off centered (in positive direction) as seen in Fig. 3.

## 4 DISCUSSIONS

The data presented in this study reflect the combined performance of the immobilization system components (mask, BoS frame, cushion, and bite block) and the motion of the robotic couch. This is especially relevant to compact systems such as the one used in this study where limited gantry motion is compensated for using more robotic couch angles compared to a 360‐degree gantry room, where coplanar beam delivery capability is readily available without the need for 180‐degree couch rotation.

### 4.A Inter‐ and intra‐fractional translational and rotational corrections

The distribution of displacements along PA and LAT directions demonstrates some directional preference. Group mean error (M_g_) along PA direction (2.3 mm) is likely because of a difference in the support and anchoring mechanism of the BoS insert between the CT simulation couch and in‐room robotic treatment couch. It is noteworthy that the reported^8^ M_g_ for a similar robotic couch used for carbon therapy is also highest along PA compared to other directions. Similarly, directional preference of distribution of pitch angle corrections is likely correlated to couch sag. For the LAT direction, the group mean error (M_g_) is 1.6 mm and it is due to a known LAT offset of the in‐room robotic couch compared to CT couch. The above error values show that we are consistently able to localize patients well within our clinical setup error margin for brain lesions of 3 mm as long as daily image guidance is used.

The obtained correctional values in translational and rotational dimensions are consistent with a previous study for a similar setup by Hsi et al.^3^ Their head and neck patients were treated on an IBA (Ion Beam Applications) system that utilizes a KUKA robotic couch with Qfix mask coupled to BoS frame. The magnitude of the translational M_g_ along SI and PA are an order of magnitude smaller than those reported for non‐robotic couches with integrated volumetric imaging solutions for daily localization.^9^ The translational systematic errors (*σ*_g_) are comparable to those reported for non‐robotic couches^9^ and conventional linear accelerators such as the Elekta Synergy MLCi accelerator with carbon fiber tables.^10^ In addition, the random errors are significantly smaller than those reported for both above systems.^9^, ^10^ In the case of rotational uncertainties, the inter‐fractional systematic and random errors are remarkably smaller than those reported for other particle therapy systems.^10^ The intra‐fractional translational and rotational uncertainties are considerably smaller than those associated with inter‐fractional motion, and are an order of magnitude smaller than those previously reported for a full gantry proton system.^11^ We find intra‐fractional uncertainty along PA direction to be higher than other directions.

### 4.B Effectiveness of bite block in conjunction with Klarity mask

Our results show that the addition of a bite block does not enhance immobilization effectiveness. Translational M_g_ and *σ*_g_ or setup errors associated with cases without bite block show smaller (better) values. On the other hand, *σ*_f_ for cases with bite block is similar to cases without one. The trends are similar for rotational uncertainties. Lack of enhancement along PA direction can be because the bite block is not directly attached to the couch as shown in Fig. 1. One might conclude that using bite blocks complicate the setup procedure and increases patient discomfort, which in turn may lead to an increase in inter‐fraction uncertainty.^12^ It is worth mentioning that while the bite block used in this study is relatively malleable, it is not considered a custom bite block that would mold specifically to patient anatomy.

### 4.C Phantom measurements

The mean of magnitude of the couch shifts along PA direction was found to be 3.3 mm from nominal position, which suggests that the robotic couch is compensating for the declining of the treatment room couch correctly. The same argument is valid about rotational corrections like the pitch. Mean of the pitch is different by 0.15° from nominal, which is caused by couch‐top sag. As for lateral shifts, we found that the distribution of lateral shift reflects a systematic difference between BoS frame position in CT room and treatment room.

## 5 CONCLUSIONS

Our study presents a comprehensive robustness evaluation of a widely used cranial immobilization systems in proton therapy and specifically in a compact system setting. We show that the BoS frame localization technique in conjunction with thermoplastic mask yields submillimeter and subdegree intra‐factional uncertainty for all translational and rotational degrees of freedom, respectively. This level of precision and reproducibility fares better or comparable to most published studies on this topic to date. In addition, we show that the use of a mask‐attached type bite block has marginal impact on inter‐ or intra‐fraction uncertainties compared to no bite block.

## ACKNOWLEDGMENTS

The authors would like to thank the radiation therapist team at Marjorie and Leonard Williams Center for Proton Therapy at Orlando Health for providing assistance with this project.

## CONFLICT OF INTEREST

The authors declare no conflict of interest.
